# Supplementary material for: MMP release following cartilage injury leads to collagen loss in intact tissue: A computational study
Source: PLoS Comput Biol. 2026 Jan 20;22(1):e1013209. doi: 10.1371/journal.pcbi.1013209 (PMC12851498; doi:10.1371/journal.pcbi.1013209)
Supplement: S1 Text — Fig A. Comparison of the injury model with and without the basal loss of collagen content. Fig B. Comparison of the biomechanical model with and without lesion geometry. Fig C. Chemical maps of collagen content. Fig D. Effect of imposing lateral gradient in the initial collagen content distribution. Fig E. Effect of imposing lateral gradient in the initial cell damage. Table A. Biochemical model parameters. (DOCX) [file pcbi.1013209.s001.docx]

**Supplementary Material – S1 Text**

**MMP release following cartilage injury leads to collagen loss in intact tissue – a computational study**

Moustafa Hamada^1,*^, Atte S.A. Eskelinen^1^, Joonas P. Kosonen^1^, Cristina Florea^1^, Alan J. Grodzinsky^2^, Petri Tanska^1,3^, Rami K. Korhonen^1^

^1^Department of Technical Physics, University of Eastern Finland, Kuopio, Finland

^2^Departments of Biological Engineering, Electrical Engineering and Computer Science, and Mechanical Engineering, Massachusetts Institute of Technology, Cambridge, Massachusetts, USA

^3^Department of Oncology, Kuopio University Hospital, Wellbeing Services County of North Savo, Kuopio, Finland.

**^*^Corresponding author at**: Department of Technical Physics, University of Eastern Finland, Yliopistonranta 8, POB 1627, 70211 Kuopio, Finland.

Email address: ***moustafa.hamada@uef.ﬁ***  Telephone number: **+358449194157**

# **Section A – Parameters in the biochemical model**

The simulated cartilage degradation consisted of the loss of both collagen and aggrecan content^1^. Both components were degraded via cell-driven mechano-inflammation: in response to injurious loading, the deformation/shearing of the cartilage tissue is associated with increased concentration of damage cells (*i.e.*, increased catabolic activity of cells)^2^. Damaged cells exhibit increased expression of proteases such as aggrecanases and matrix metalloproteinases (MMP). MMPs are primarily responsible for the collagen fibrils degradation, while aggrecanases are linked with aggrecan loss^3–6^. Changes in MMP and intact collagen concentrations are described in Equations (9-11). The degraded collagen concentration is given by:

| $\frac{\partial C_{\mathrm{cold}}}{\partial t}=f_{agg,protect}\left( C_{\mathrm{agg}} \right)k_{mmp,catalytic} C_{\mathrm{mmp}}\left( \frac{C_{\mathrm{col}}}{C_{\mathrm{col}}+K_{m,mmp}} \right)+k_{\mathrm{basal}}C_{\mathrm{cold}},$ | (Eq. S1) |
| --- | --- |

The change in aggrecanase concentration $C_{\mathrm{aga}}$ in the tissue is described as:

| $\frac{\partial C_{\mathrm{aga}}}{\partial t}=D_{\mathrm{aga}}\nabla^{2}C_{\mathrm{aga}}+k_{rate,aga}S_{\mathrm{aga}}-k_{loss,aga}C_{\mathrm{aga}},$ | (Eq. S2) |
| --- | --- |

where $k_{rate,aga}$ represents rate constant of generating aggrecanases from damaged cells and $k_{loss,aga}$ represents the rate of aggrecanases degradation.

The change in aggrecan concentration $C_{\mathrm{agg}}$ is defined as:

| $\frac{\partial C_{\mathrm{agg}}}{\partial t}=D_{\mathrm{agg}}\nabla^{2}C_{\mathrm{agg}}+R_{\mathrm{biosynthesis}}C_{\mathrm{cell}}\left( \frac{C_{\mathrm{agg}}}{C_{\mathrm{tar}}} \right)-k_{aga,catalytic}C_{\mathrm{agg}}\left( \frac{C_{\mathrm{agg}}}{C_{\mathrm{agg}}+K_{m,aga}} \right),$ | (Eq. S3) |
| --- | --- |

where $C_{\mathrm{tar}}$ represents the target (homeostatic) aggrecan concentration at which the biosynthesis of new aggrecan ceases to increase, $k_{aga,catalytic}$ represents rate of catalytic activity of aggrecanases, and $K_{m,aga}$ is Michaelis constant for aggrecanases binding to aggrecan. The $R_{\mathrm{biosynthesis}}$ is describing the depth-wise rate of aggrecan production:

| $R_{\mathrm{biosynthesis}}=P_{\mathrm{agg}}\left( 1+\frac{0.9(1-z)}{H} \right),$ | (Eq. S4) |
| --- | --- |

where $P_{\mathrm{agg}}$ is the basal amount of aggrecan produced by the healthy cells, $z$ is the normalized axial co-ordinate within the tissue (*z* = 0 at the surface, *z* = 1 at the bottom), and $H$ is the thickness of the tissue. The change in degraded aggrecan concentration was governed by:

| $\frac{\partial C_{\mathrm{aggd}}}{\partial t}=D_{\mathrm{aggd}}\nabla^{2}C_{\mathrm{aggd}}+k_{aga,catalytic}C_{\mathrm{agg}}\left( \frac{C_{\mathrm{agg}}}{C_{\mathrm{agg}}+K_{m,aga}} \right) ,$ | (Eq. S5) |
| --- | --- |

The values used for the model parameters are detailed in Table A.

# **Section B – Injury model without the basal loss**

In experimental results, the free-swelling control group showed a significant decrease on day 12 in collagen content throughout the entire depth of the tissue compared to day 0 (Fig 2C in main text). We wanted to represent this effect in the model, to be able to differentiate between collagen loss in the free-swelling culture conditions and collagen loss caused by MMP-related enzymatic cleavage. Without the basal loss term $k_{\mathrm{basal}}C_{col}$ in Equation (11), the injury model shows bulk collagen loss of 13% on average (±7%) by day 12 (Fig AI). On the other hand, when $k_{\mathrm{basal}}$ is incorporated, the simulations showed an average of 30% (±7%) collagen loss (Fig AII), representing more accurate match with the experimental results (~35% average bulk decrease in collagen content on day 12 compared to day 0).

| Model parameter | Value | Description | Reference |
| --- | --- | --- | --- |
| $C_{cell,healthy} (cells.m^{-3})$ | 1.5 × 10^14^ | Initial healthy cell concentration **(Eq 6)** | ^6^ |
| $k_{\mathrm{INJ}} (-)$ | 0.35 | Maximum allowed cell damage **(Eq 6)** | ^2^ |
| $k_{\mathrm{mmp}}$  (10^-21^ mol) | 0.25 | Production of MMPs from damaged cells **(Eq 8)** | Model fit |
| $k_{rate, mmp}$  (${10}^{-5}s^{-1}$) | 3.6 | MMPs production rate **(Eq 9)** | Model fit, ^6^ |
| $k_{mmp,cold}\left( m^{3}{\cdot mol}^{-1}\cdot s^{-1} \right)$ | 4.7 ×10^-4^ | Rate of MMP binding to degraded collagen **(Eq 9)** | ^6^ |
| $n_{R,cold}(-)$ | 320 | Number of MMP binding sites on degraded collagen **(Eq 9)** | ^6^ |
| $k_{\mathrm{act}} ({10}^{-4}mol\cdot m^{-3})$ | 3 | Aggrecan concentration at half maximum of MMP activity **(Eq 10)** | ^6^ |
| $n (-)$ | 6 | Hill coefficient of MMP activity **(Eq 10)** | ^6^ |
| $k_{mmp,catalytic} {(s}^{-1})$ | 1.5 | Catalytic activity rate of MMPs with collagen fibrils **(Eq 11)** | ^6^ |
| $K_{m,mmp} (mol\cdot m^{-3})$ | 0.0021 | Michaelis constant for MMPs **(Eq 11)** | ^6^ |
| $k_{\mathrm{basal}} ({10}^{-7}s^{-1})$ | 2 | Rate of basal collagen loss **(Eq 11)** | Model fit |
| $k_{rate,aga} ({10}^{-5}s^{-1})$ | 3.6 | Rate constant for generating aggrecanases from damaged cells **(Eq. S2)** | ^6^ |
| $k_{loss,aga}({10}^{-4}s^{-1})$ | 1 | Aggrecanase degradation rate **(Eq. S2)** | ^6^ |
| $P_{\mathrm{agg}}({10}^{-22}\mathrm{mol}s^{-1})$ | 2.4 | Basal amount of aggrecan production **(Eq. S4)** | ^6^ |
| $C_{\mathrm{tar}}(mol\cdot m^{-3})$ | 0.011635 | Target aggrecan concentration **(Eq. S3)** | Model fit, ^6^ |
| $k_{aga,catalytic}{(s}^{-1})$ | 0.9 | Catalytic activity rate of aggrecanases with aggrecan **(Eq. S5)** | ^6^ |
| $K_{m,aga}({10}^{-5}mol\cdot m^{-3})$ | 5 | Michaelis constant for aggrecanases **(Eq. S5)** | ^6^ |

**Table A. Biochemical model parameters**


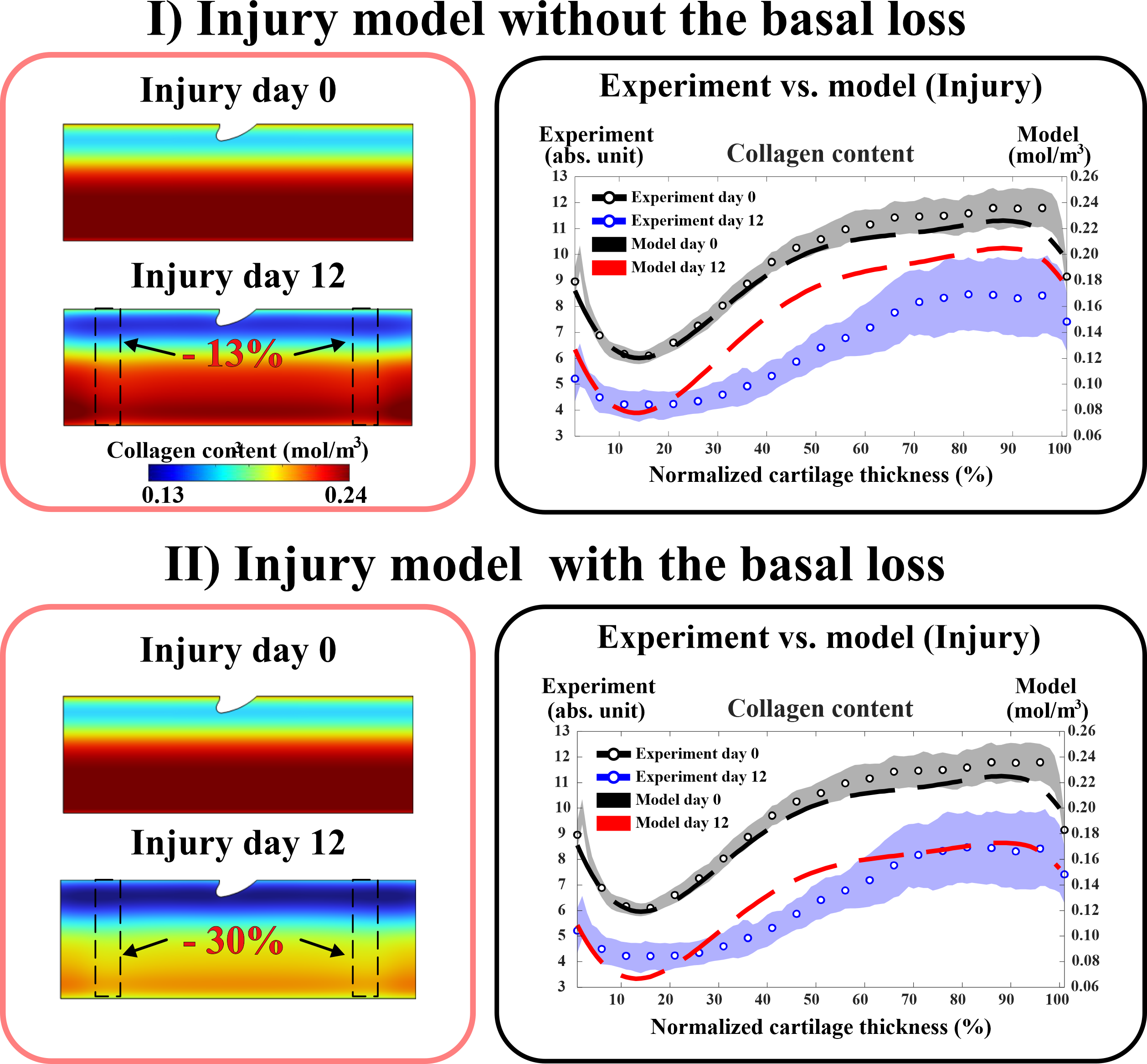


**Fig A. Comparison of the injury model with and without the basal loss of collagen content. I)** Injury model without the basal loss showed 13% bulk decrease in collagen content by day 12 (vs. 35% decrease in the experiment), **II)** Injury model with the basal loss showed 30% bulk decrease in collagen content by day 12.

# **Section C – Considering lesion geometry in the biomechanical model**

Our model results did not show any localized decrease in collagen content near cartilage lesion. The model did not incorporate lesion formation and therefore it assumed that lesion is formed directly after the loading. Accordingly, maximum shear strains were not localized at the cartilage surface and were more excessive in deeper regions of the tissue. We hypothesize that if lesion was formed during the loading, this could lead to localized excessive shear strain around the lesion which could lead to higher cell damage and MMP-driven catabolic activity around this region compared to intact regions, contributing to the excessive localized decrease in collagen content observed in our experimental work^7^. We wanted to test this hypothesis and implemented a biomechanical model when the loading is applied on cartilage geometry with and without lesion. Single cycle of unconfined compression (20% strain and 100%/s strain rate) was applied in both cases (Fig B). For the model utilizing lesion geometry, same boundary conditions and material properties were applied as in model with intact geometry (see Section 2.3 in main text) and the cartilage geometry was meshed using 918 linear quadrilateral elements of type CPE4P.

When comparing the maximum shear strain values at 20% of compressive strain, the model with lesion geometry exhibited excessive localized shear strain of 160% around the lesion compared to intact regions away from lesion that showed shear strains of less than 50%. The model with intact geometry exhibited shear strains no more than 40%. The localized shear strains suggest that cell damage could be localized near the lesion potentially causing more catabolic enzymatic activity of MMPs around the lesion compared to regions of the cartilage with no lesions.


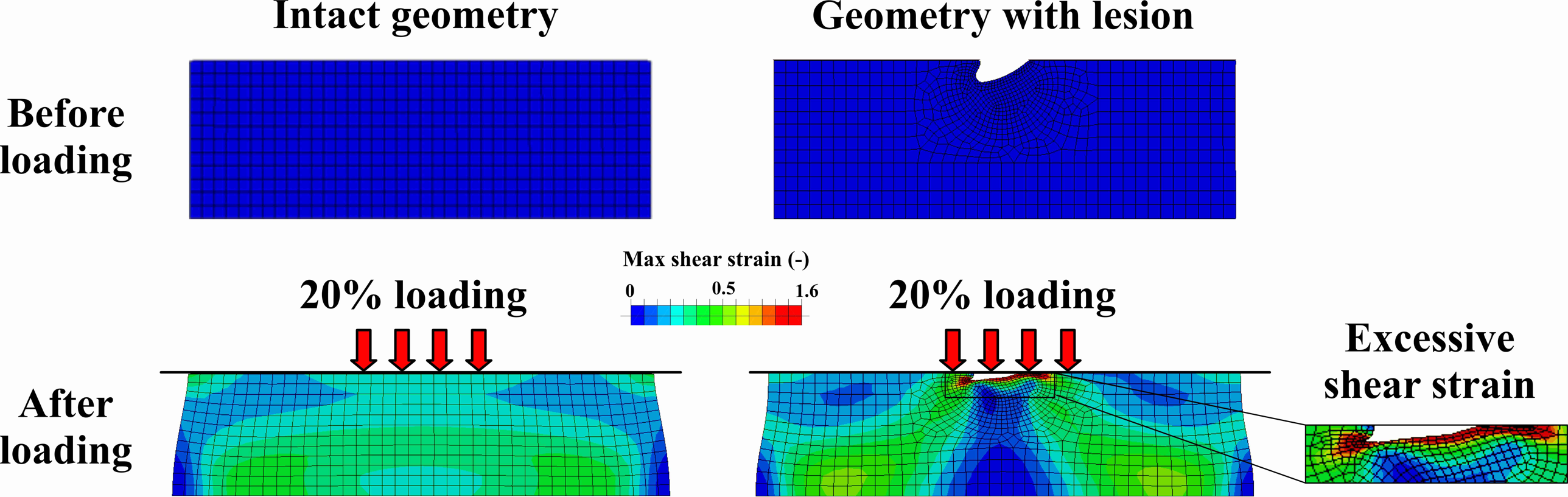


**Fig B. Comparison of the biomechanical model with and without lesion geometry.** After applying single load-unload cycle of unconfined compression (20% strain, 100%/s strain rate), cartilage with lesion geometry exhibited excessive localized shear strain near lesion (~160% shear strain) compared to intact regions (less than 50% shear strain).

# **Section D – Considering lateral gradient in collagen distribution**

Experimental samples exhibited stochastic, sample-specific x-axis variation in collagen content (Figure C). This variability likely arises from both biological factors (e.g., different knees or anatomical locations) and technical factors related to tissue preparation and loading condition (e.g., small differences in sample geometry or plate-tissue alignment). Such factors may influence the local mechanical response and in turn the ultimate day-12 collagen loss distribution. In contrast, our model assumes collagen to be laterally homogeneous and depth-dependent only, because accurately capturing the sample-specific lateral discontinuities would require individualized model variables characterization.

To evaluate whether considering x- direction as variable would affect our model outcomes, we performed two sensitivity analysis in which x-axis gradients were introduced into key variables (initial collagen content, and cell damage) using the most laterally heterogeneous sample from day 0 as a reference. For each analysis, collagen loss was quantified in four rectangular regions spanning depth (y-axis) and lateral (x-axis) directions.

## **Lateral variation in initial collagen content**

We incorporated a linear lateral gradient in the initial collagen content distribution (~20% points higher collagen content on the right compared to the left). In the mechanical model, collagen is represented as a normalized density fraction (reflecting fibril mechanical stiffness), while in the biochemical model, the same collagen network is represented as a molar concentration (subject to enzymatic degradation).

Introducing this gradient had no effect on the mechanical response: maximum shear strain remained nearly identical to the homogeneous case (Figure D), and therefore the distribution of damaged cells also remained laterally uniform. This indicates that a 20% lateral variation in collagen content is insufficient to alter shear strain under the high rate loading applied, where the response is more strongly influenced by collagen fibril orientation and fluid pressurization.

Nevertheless, the imposed gradient produced minor lateral differences in day-12 collagen loss. Regions with initially lower collagen content showed slightly greater loss, consistent with reduced MMP binding to degraded collagen in low-collagen regions, leaving more free MMPs available to degrade intact collagen.

## **Lateral variation in initial cell damage**

We next examined a hypothetical extreme scenario by imposing a 20% lateral gradient in initial cell damage, representing a case where shear strain or platen–sample contact would be laterally uneven (Figure E). This resulted in small lateral differences in collagen loss: ~6% greater loss in the upper right region compared with the upper left, and ~3% greater loss in the lower right. These changes reflect local increases in MMP production in regions with higher cell damage.

Across both analyses, incorporating lateral variation into either the initial collagen content or the initial cell damage produced only minor lateral differences in day-12 collagen loss. These effects were far smaller than the ~20% lateral discontinuities observed experimentally, which vary in both magnitude and pattern between samples. Accurately reproducing such behavior would therefore require sample-specific models that explicitly represent individualized x-axis variability in cartilage composition and structure which is outside the scope of the current study. Thus, a depth-dependent modeling framework remains appropriate for addressing the central mechanobiological hypothesis of this work.


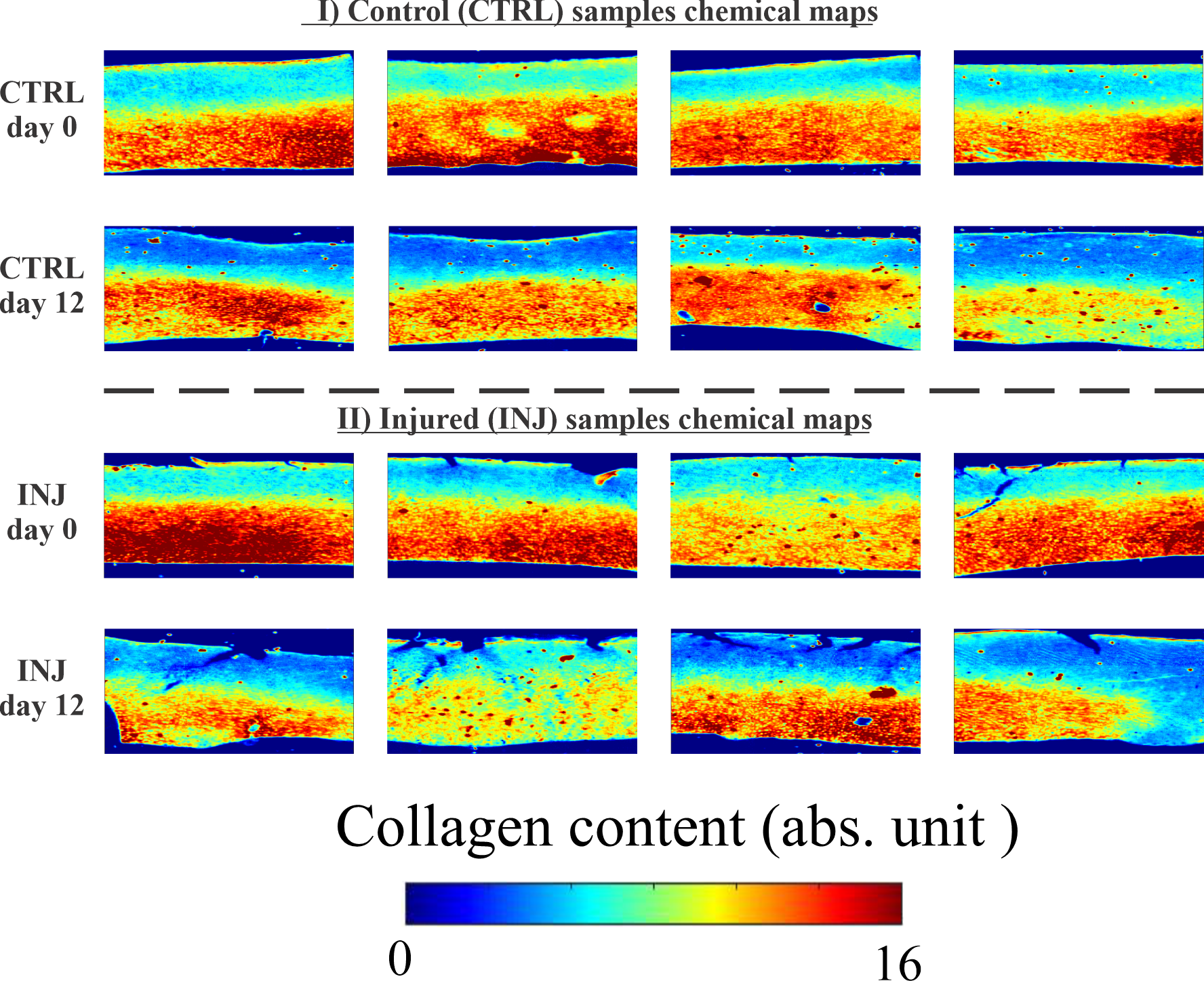


**Fig C. Chemical maps of collagen content for** I) Control non-injured group (CTRL) and II) Injured group (INJ) both on day 0 and day 12. Maps on day 12 in both groups shows discontinuous collagen content in the lateral (x-axis) direction between samples. However, collagen content along the depth (y-axis) consistently exhibits a clear depth-dependent gradient pattern.


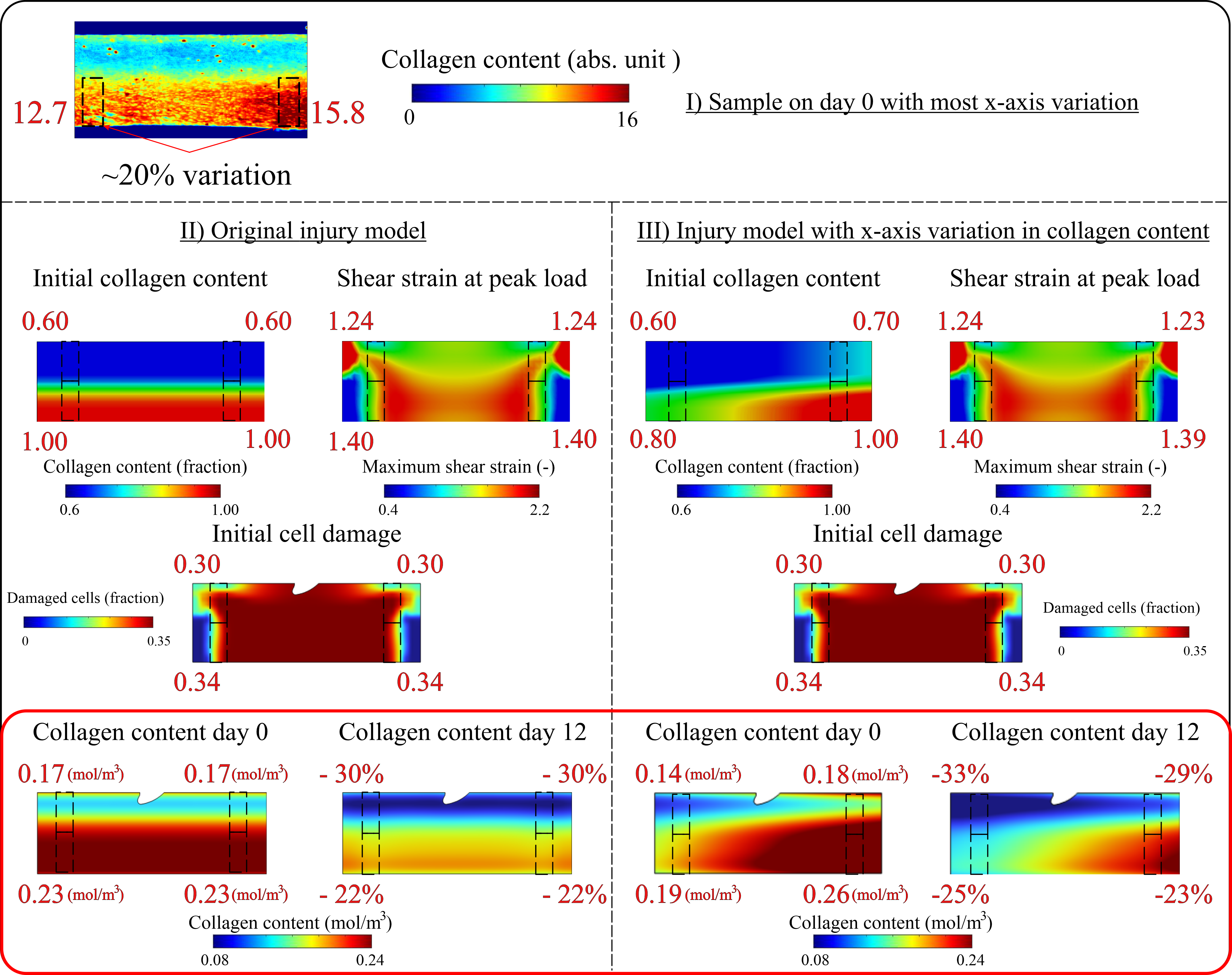


**Fig D. Effect of imposing lateral gradient in the initial collagen content distribution.** In the mechanical model, collagen content is represented as a normalized density fraction relative to the maximum collagen content (reflecting fibril stiffness), whereas in the biochemical model, it is represented as a molar concentration subject to enzymatic degradation.

**Fig E. Effect of imposing lateral gradient in the initial cell damage.**


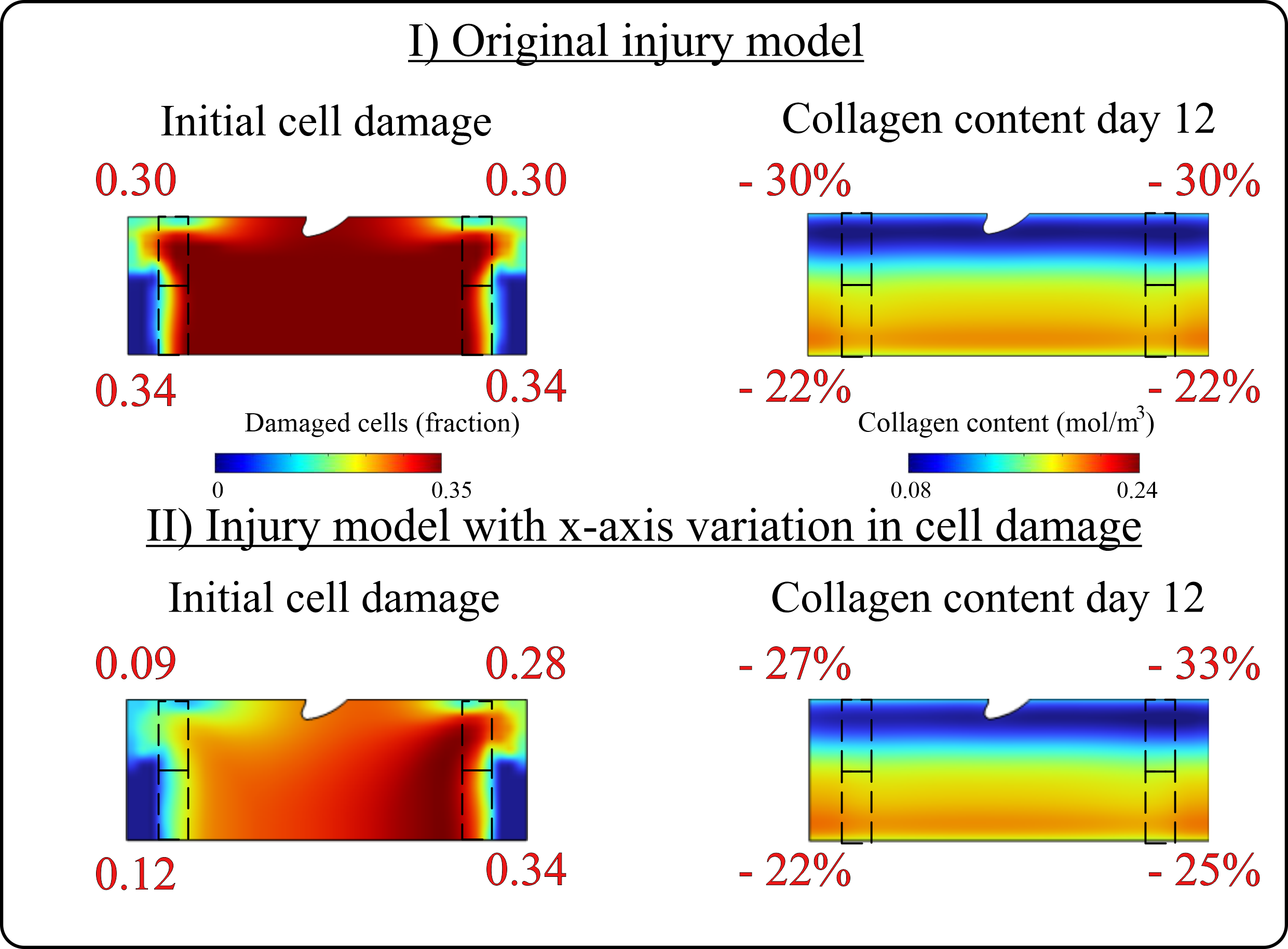


**References**

1. Sophia Fox, A. J., Bedi, A., Rodeo, S. A. The basic science of articular cartilage: Structure, composition, and function. *Sports Health* **1**, 461–468 (2009).

2. Loening, A. M. *et al.* Injurious mechanical compression of bovine articular cartilage induces chondrocyte apoptosis. *Arch Biochem Biophys* **381**, 205–212 (2000).

3. Leong, D. J. *et al.* Matrix metalloproteinase-3 in articular cartilage is upregulated by joint immobilization and suppressed by passive joint motion. *Matrix Biology* **29**, 420–426 (2010).

4. Sun, H. Bin, Nalim, R., Yokota, H. Expression and activities of matrix metalloproteinases under oscillatory shear in IL-1-stimulated synovial cells. *Connective Tissue Research* **44** 42–49 (2003).

5. van Meurs, J. *et al.* Active Matrix Metalloproteinases Are Present in Cartilage During Immune Complex-Mediated Arthritis: A Pivotal Role for Stromelysin-1 in Cartilage Destruction. *The Journal of Immunology* **163**, 5633–5639 (1999).

6. Kar, S. *et al.* Modeling IL-1 induced degradation of articular cartilage. *Arch Biochem Biophys* **594**, 37–53 (2016).

7. Hamada, M. *et al.* Loss of collagen content is localized near cartilage lesions on the day of injurious loading and intensified on day 12. *Journal of Orthopaedic Research* (2024).

8. Jönsson, V. *et al.* Influence of articular cartilage sample geometry on mechanical response and properties using finite element simulation. *J Biomech* **176**, (2024).
