# Supplementary material for: MMP release following cartilage injury leads to collagen loss in intact tissue: A computational study
Source: PLoS Comput Biol. 2026 Jan 20;22(1):e1013209. doi: 10.1371/journal.pcbi.1013209 (PMC12851498; doi:10.1371/journal.pcbi.1013209)
Supplement: Attachment — Submitted filename: Response to reviewers.docx [file pcbi.1013209.s002.docx]

**Manuscript number: PCOMPBIOL-D-25-01150, revision number: 1**

***RESPONSES TO REVIEWER #1 COMMENTS:***

**General comment:** Hamada’s work presents a finite element–based mechano-signaling model that mechanistically links acute mechanical injury to biochemical collagen degradation in articular cartilage. The model effectively captures key causal sequences: mechanical insult → cell damage → MMP upregulation → collagen degradation, which provides mechanistic insight that bridges the gap between mechanical trauma and subsequent matrix breakdown. Notably, authors validate their simulations against ex vivo data from injured bovine cartilage explants cultured for 12 days. They demonstrated strong concordance with model predictions (~30% simulated vs. ~35% experimental collagen loss), thereby reinforcing the biological plausibility of the proposed mechanism.

This study delivers a compelling and well-validated mechano-signaling model that advances the field of computational cartilage degeneration. Its integration of mechanical strain, cell viability, and enzyme-mediated collagen degradation distinguishes it from prior frameworks. The methodology is rigorous, the model is biologically grounded, and the presentation is clear and well-illustrated. Regardless of the novelty, clarity and the promising results presented by this manuscript, I have found that the authors may need to pay attention to the following issues.

**Authors’ response:** We thank the reviewer for taking the time to review the manuscript and for the positive feedback. The issues highlighted by the reviewer are discussed below. The necessary actions needed, and changes made in the revised manuscript text are highlighted with yellow.

**Reviewer comment #1:** Please clarify how the proposed finite element mechano-signaling framework improves upon or differs from earlier cartilage degeneration models, particularly those incorporating mechanical and biochemical factors.

**Authors’ response:** To the best of our knowledge, our proposed model is the first to couple mechanically induced cell damage and subsequent MMP activity to explain collagen degradation in cartilage particularly post-injury. We are also accounting for the depth-dependent distribution of collagen content and its loss temporally, supported by experimental data measured with Fourier transform infrared (FTIR) microspectroscopy.

In previous finite element (FE) cartilage models, collagen degradation has typically been represented through fibril degeneration or fibril network softening driven by excessive mechanical loading, such as high collagen fibril strains or maximum principal (tensile) stresses in cartilage tissue^1–4^, or through collagen fibril reorientation^5^. Conversely, models addressing biochemically driven or inflammation-driven mechanisms have generally described collagen loss as a bulk process or incorporated the depth-dependency through variations in collagenase activity^6,7^. We are highlighting the novelty in our work more clearly in the revised manuscript.

**Authors’ actions:**

*Abstract* – *(page 2, lines 21–25):* “Collagen damage in articular cartilage plays a key role in post-traumatic osteoarthritis, but the underlying mechanobiological pathways leading to collagen fibril degeneration after injury remain incompletely understood. We hypothesized that mechanical injurious loading induces localized cellular damage in cartilage, which in turn triggers the release of collagen-degrading matrix metalloproteinases (MMPs) and depth-wise collagen loss”

*Introduction* – *(pages 5–6, lines 106–108):* “Mechanobiological models offer a promising avenue for investigating the driving mechanisms behind early PTOA-related compositional changes, which are challenging to attain solely by experiments^7–10^. Regarding collagen damage, a major focus of current modeling studies is typically on the collagen mechanics such as collagen network softening/damage driven by excessive tensile stresses in the cartilage matrix or tensile strains in the individual collagen fibrils^2,11^. On the other hand, several computational studies have addressed enzymatic activity and biochemical signaling caused by traumatic joint injury that could lead to the loss of collagen fibrils^6,12–14^. However, these models have not incorporated cell damage-driven MMP-activity post-injury which contributes to the localization and temporal progression of collagen loss in cartilage^7,8,14^.”

*Discussion* – *(page 24, lines 428–430):* “In conclusion, our model replicates experimental collagen loss after injury through injury-induced mechano-signaling in cartilage cells, leading to downstream MMP activity and subsequent early depth-wise collagen loss.”

**Reviewer comment #2:** Why were only day 0 and day 12 selected for experimental validation? Inclusion of intermediate time points (e.g., day 3, 6, or 9) would improve temporal resolution and model calibration.

**Authors’ response:** We thank the reviewer for raising this important point. We fully agree that including additional time points would improve the temporal resolution and strengthen model calibration. Intermediate time points before day 12 could reveal the onset of collagen loss and improve our understanding of the temporal progression of MMP upregulation and catabolic activity in response to injury-driven cell damage. However, at this stage, experimental data at earlier time-points have not yet been analyzed. As part of our ongoing work, we plan to incorporate intermediate time points (i.e., days 3 and 7) to improve model calibration in its future iterations.

**Authors’ actions:**

*Discussion* – *(pages 22-23, lines 398–403):* “… Therefore, the experimental findings and model parameter values presented here might not be directly translatable to human cartilage without further validation. In addition, the collagen content experimental data used in this work for comparison were available only at two time points (i.e., day 0 and day 12). Incorporating intermediate time points in future work would enable a more accurate characterization of the temporal progression of MMP upregulation and catabolic activity following injury-induced cell damage and thereby allow a more precise temporal assessment of collagen loss over the 12-days period.”

**Reviewer comment #3:** Please explain the rationale for selecting ±50% parameter variation. Does this reflect biological uncertainty, empirical bounds, or a conventional modeling assumption? Were alternative distributions (e.g., uniform or log-normal) considered?

**Authors’ response:** The ±50% parameter variation was a conventional modeling assumption. This range was determined through model calibration and preliminary simulations since no experimental data were available to define their quantitative ranges or uncertainty bounds. Hence, alternative distributions and probabilistic sensitivity analysis are not feasible. The uniform ±50% variation was applied systematically to the parameters used in the analysis to quantitatively estimate sensitivity of collagen content loss to the selected parameters.

**Authors’ actions:**

*Methods* – *(page 15, lines 272–278):* “Reference values for these parameters were selected based on preliminary simulations (Table 2), in which the simulated depth-wise collagen content was matched with the experimentally measured data^15^. For all parameters, a uniform ±50% range of values was applied to systematically assess the sensitivity of collagen content to changes in each parameter. This ±50% range represents a conventional modeling assumption based on preliminary calibration, given the absence of direct experimental measurements for these parameters. During the preliminary calibration, we observed that $k_{\mathrm{act}}$ has a limited influence on collagen loss under the current aggrecan concentration used in the model. Therefore, we further evaluated its effect by increasing the value by a factor of 100.”

**Reviewer comment #4:** Figure 1 is somewhat blurred and visually dense. The meaning of “ABAQUS” in panel (b) is not clear. Additionally, the use of bright color scales and large arrows reduces visual clarity and focus.

**Authors’ response and actions:** We appreciate the reviewer’s feedback. In the revised manuscript, we improved the overall visual clarity of the figure by adjusting the used color scales and reducing the arrows and the texts. The ABAQUS and COMSOL logos were also removed.

**Reviewer comment #5:** Figure labeling and affiliations:

o Panels should be labeled with uppercase letters (A, B, C…) in the top-left corner, following journal style.

o Author affiliations should use superscript numbers (1, 2, 3…), rather than lowercase letters (a, b, c…).

**Authors’ response and actions:**

All figures’ panels have been relabeled using upper case letters, consistent with the journal’s formatting guidelines. Author affiliations have also been updated to use superscript numerical identifiers.

***RESPONSES TO REVIEWER #2 COMMENTS:***

**General comment:** Hamada et al. report in this manuscript a computational model for mechanical stress-induced cartilage damage. Their model is based on the authors’ studies on injurious cartilage tissues including the use of Fourier transform infrared microspectroscopy (FTIR) as reported earlier this year. Thus, the technical and analytical aspects as well as possible involvements of matrix metalloproteinases are not new here. The main novelty lies in computational modeling. The authors’ efforts to build a predictive model are important for future clinical applications and the development of therapeutics for osteoarthritis. It is noteworthy that the primary driver of collagen loss is attributed to the release of catabolic enzymes such as MMPs triggered by force-induced cell damage in the cartilage. Although modeling a complex system may become challenging, the authors need to address several important issues described below.

**Authors’ response:** We thank the reviewer for the insightful feedback. Author’s responses and actions considered to address reviewer comments in the revised manuscript text are highlighted below.

**Reviewer comment #1:** I am concerned that their experimental data acquired in FTIR may not be fully utilized for modeling. In the Results section, page 16, lines 298-302, while the authors describe a result from computational simulations along with experimental chemical maps (Figs 2a and 2b), it is unclear about the reason why the area highlighted by a rectangular strip (dotted line) as an ROI (region of interest) was chosen for comparisons. The chemical/experimental maps on day 12 for either control or injury groups show highly variable and discontinuous shades of collagen distributions along the x-axis. In contrast, simulation maps show uniform distributions along the x-axis, which is probably not considered as a variable. In Methods, the authors describe that “two full-depth and 200 μm wide regions of interest (ROI) were defined in each histological section” (line 136), providing the dimension of a ROI, but not how ROIs are selected. Thus, the quantification described in Results means little. The ROI criteria should be based on objective selections of measurable parameters. The authors also need to clarify how ROIs can capture the discontinuous nature of the samples as found even in non-injurious control. Since the authors attempt to model the effect of diffusible factors generated by injurious cells in the cartilage, I feel that this is a critical issue for the authors to address properly for modeling. In the current analytical model, the chemical model is being treated as if it was a 1D model. The biomechanical and high shear strain model offers information in 2D to model the shearing force that presumably triggers cell damage and distributions of diffusible factors in a 2D plane. Therefore, there is a discrepancy between dimensions while tissue collagen contents are discontinuously localized in 2D in day-12 cartilage samples.

The issue raises the concern that the current modeling workflow appears premature in collagen distribution data interpretation. The model seems overly simplified without sufficient justification. It could be significantly strengthened by reconsidering how these special complexities are integrated.

**Authors’ response:** We will address the reviewer’s comment in two parts. First, we will clarify **the criteria used for selecting the regions of interest (ROIs)** and explain how these selections capture the discontinuous nature of collagen content in the samples. Second, we will address **the effect of considering lateral gradient in collagen content distribution into our models** (derived from one of our samples) and analyze its impact on the model results.

1) **The choice of ROIs in the experiment:**

Two regions of interest (ROIs) were selected to encompass the full tissue thickness (exhibiting the predominant variation in collagen content occurring on y-axis) in intact areas positioned midway between the lesion edge and the section edge (Figure 1). This ensured that the analyzed regions were unaffected by the potential artifacts around the lesion and the sample edges. Each ROI was 200-µm wide and positioned on opposite sides of the lesions to capture the lateral stochastic variation in collagen content. Within each ROI, values were averaged along the x-axis, minimizing the influence of this discontinuity in collagen content.

The lateral discontinuities observed in the collagen maps (Figure 2), particularly pronounced by day 12, may arise from biological factors (e.g., samples from different knees and anatomical locations), as well as technical experimental factors (e.g., minor differences in sample geometry or imperfect tissue–plate contact during loading). Selecting two separate ROIs per histological section therefore ensured: a) consistent inclusion of intact regions (i.e., away from any lesions) across all samples and b) effective averaging of stochastic lateral heterogeneity within each section. The initial collagen distribution used in the model was defined using the average depth-dependent collagen content profile of samples on day 0. Figure 2 illustrates the lateral variability in collagen content observed across different samples in different groups.

**Figure 1. Two regions of interest of 200 µm width selected from each 3 mm cartilage section.**

**Figure 2. Chemical maps of collagen content for A) Control non-injured group (CTRL) and B) Injured group (INJ) both on day 0 and day 12.** Maps on day 12 in both groups shows discontinuous collagen content in the lateral (x-axis) direction between samples. However, collagen content along the depth (y-axis) consistently exhibits a clear depth-dependent gradient pattern.

2) **Considering potential sources of lateral variation in the collagen content in the model:**

The non-uniform x-axis distribution of collagen content is a crucial point raised by the reviewer. We used our model to examine whether key model variables would influence this discontinuity pattern when allowed to vary along the x-axis and whether this lateral variation would have an impact on model outcome. Accordingly, we performed **two additional sensitivity analyses** (Figure 3&4), introducing x-axis variation into:

1. The initial collagen content distribution
2. The initial cell damage distribution after injurious loading

Our results show that (1) **introducing a lateral gradient in the initial collagen content did not affect the shear strain distribution or the resulting cell damage** compared to the original model (Figure 3). It produced only ~4% lateral variation in collagen loss on day 12, far smaller than the ~20% lateral discontinuities observed experimentally, which also differ in pattern and magnitude between samples. (2) **Introducing a lateral gradient in initial cell damage resulted in similarly small variation** (3% to 6% lateral variation, Figure 4).

These findings indicate that reproducing the experimentally observed sample-specific x-axis heterogeneity requires individualized model variables which are beyond the scope of this work. Therefore, focusing on the predominant depth-dependent variation remains appropriate for addressing the central mechano-signaling hypothesis of this study. Detailed descriptions of the sensitivity analysis and results are provided below.

**a) Lateral variation in initial collagen content:**

We incorporated a linear lateral gradient in the initial collagen content (~20% higher on the right compared to the left). Since shear strain is a primary driver of the MMP-mediated collagen loss in our framework, this analysis examined whether such lateral heterogeneity would alter the mechanical response and thereby influence cell damage and day-12 collagen loss. In our framework, the same collagen fibril network is represented in two complementary ways: as a normalized density fraction relative to the maximum collagen content in the mechanical model (reflecting fibril mechanical stiffness) and as a molar concentration in the biochemical model (subject to enzymatic degradation).

To evaluate the lateral differences, shear strain, cell damage, and day-12 collagen loss was quantified as average in each of four distinct rectangular regions spanning both axial and lateral directions. Applying the lateral gradient had no effect on the mechanical response: maximum shear strain remained nearly identical to the original model (Figure 3B&C), and cell damage therefore remained laterally uniform. This indicates that a ~20% lateral variation in initial collagen content is insufficient to alter the shear strain under the high-rate excessive loading applied (50% strain, 100%/s), where the mechanical response is likely dominated by other factors such as collagen fibrils orientation and fluid pressurization.

Nevertheless, in the biochemical model the imposed gradient produced minor differences in collagen loss on day 12. Regions with lower initial collagen content exhibited greater loss by day 12 (33% loss in the upper left vs. 29% loss in the upper right, Figure 3C), and these lateral differences remained minor compared with the predominant systematic depth-dependent variation along the y-axis. This lateral variation arises because low-collagen regions also contain less degraded collagen, reducing MMP binding to degraded collagen and leaving a larger fraction of free MMPs available to degrade intact collagen.

**Figure 3. Effect of imposing lateral gradient in the initial collagen content.** In the mechanical model, collagen content is represented as a normalized density fraction relative to the maximum collagen content (reflecting fibril stiffness), whereas in the biochemical model, it is represented as a molar concentration subject to enzymatic degradation.

**b) Lateral variation in cell damage:**

To further evaluate the potential influence of x-axis variation, we also introduced a lateral gradient in cell damage (~20%-points higher cell damage on the right compared to the left), representing a hypothetical extreme scenario in which injury-induced cell damage is laterally heterogeneous. This analysis evaluated whether such variation in a key driver of MMP release would influence the lateral pattern of day-12 collagen loss. The resulting differences remained small: 6% greater collagen loss in the upper right region compared with the upper left (Figure 4C) and ~3% greater loss in the lower right compared to the lower left. These differences reflect locally elevated MMP levels in regions with higher cell damage. However, the resulting lateral variation remained far smaller than ~20% lateral discontinuities observed experimentally and had only a minor effect on model outcomes compared with the predominant, systematic depth-dependent gradient.

**Figure 4. Effect of imposing lateral gradient in the initial cell damage**

Therefore, based on the conducted sensitivity analysis, incorporating lateral variation in initial collagen content or initial cell damage produced only minor lateral variation in day-12 collagen content. This suggests that the mechano-signaling mechanism proposed here is unlikely to be a driver of the pronounced lateral discontinuities observed experimentally. Instead, these discontinuities are stochastic and sample-specific. Reproducing such lateral heterogeneity would require sample-specific modeling that account for individualized x-axis variation in cartilage composition and structure, which was beyond the scope of the present study.

**Authors’ actions:**

*Methods* – *(page 7, lines 137–141):* “Next, two full-depth and 200 µm wide regions of interest (ROI) were defined in each histological section. Collagen content was extracted from these ROIs, averaged perpendicular to depth, and normalized depth-dependently (0% = surface, 100% = bottom) to obtain depth-wise collagen content profile for each explant. The ROIs were selected in intact areas located midway between the lesion and the section edges to ensure that the analyzed regions were unaffected by either the lesion or potential artifacts near the sample edges. A 200-µm width was chosen to capture the dominant depth-dependent gradient in collagen content while accounting for stochastic lateral variation in each section by averaging collagen content values across the 200-µm span.”

*Methods* – *(page 9, lines 165-166):* “The initial depth-wise collagen fraction was implemented in the model based on the mean depth-wise collagen content in intact cartilage explants at day 0 (Table 1)^15^. Likewise, the depth-wise fixed charge density (FCD, assumed to correspond the aggrecan content) distribution was implemented using optical density measurements from the same cartilage explants (characterized in^17^) and converted into FCD values as done previously^18^. The fluid fraction was obtained from literature^18^. All distributions were defined as depth-dependent while lateral direction was assumed to remain homogeneous.”

*Methods* – *(page 15, lines 279-282):* “In addition, to address the experimentally observed lateral heterogeneity in collagen content distribution, separate sensitivity analyses were conducted in which lateral variation in the initial collagen content distribution was introduced into the model. The methods and results of these analyses are described in detail in Section S4 of the Supplementary material.”

*Results*– *(page 18, lines 318-319):* “Results reflecting the effect of lateral variation in collagen content distribution are presented in the Supplementary material (Figures S4-S5).”

*Discussion*– *(pages 23-24, lines 415–422):* “It is worth noting that the model accounted for the depth-dependent distribution of collagen content in cartilage, while a homogeneous distribution was assumed along the lateral direction. Experimentally, however, the samples exhibited stochastic and sample-specific lateral variation in collagen content distribution (Figure S3). Accurately representing this variability would require sample-specific modeling, which was beyond the scope of this work. Nevertheless, when we introduced lateral variation in the initial collagen content distribution of the model, utilizing the sample with the largest x-axis heterogeneity (Figures S4-S5 in the supplementary material), this variation had a minor effect on collagen loss.”

**Reviewer comment #2 and #3:** The authors state that they did not model mechanical injuries from force, but “lesion geometry” was added to the injury model. Such lesions were based on “visual” assessments, however. If the extent (area size, shape, depth, etc.) has any impact on the injury model construction, it is highly desirable to use more objective means to identify the lesion on the images since “deformation” is indeed considered in the biomechanical continuum.

Another layer of the issue above is that force-induced “deformation” assumes no loss of material or change in lesion geometry over time in their model. The authors include discussion of previously observed lesion-associated rapid loss of collagen contents along authors’ simulations shown in Fig S2. Therefore, the authors need to consider the possibility that lesion geometry may not be static during the course of experiment. Evaluation of the shear strain that Day 12 samples initially received may be complicated by this difficulty to estimate the initial lesion geometry.

**Author’s response:** The mechanical model assumes an initially intact tissue geometry, consistent with the experimental setup in which the lesion forms during the one-second injurious loading event. In this experiment, no further mechanical loading was applied after the injurious loading event and therefore no mechanically induced lesion propagation was expected to occur during the subsequent 12-day incubation period. After simulating the injurious loading, the lesion geometry (segmented from a histological cartilage section) was incorporated only in the biochemical model, to assess whether its presence could affect the diffusivity of molecular species (e.g., MMPs, aggrecan) within the tissue.

Because the injurious loading was applied only once at the beginning of the experiment, the lesion was assumed to remain largely unchanged during the 12-day incubation period as noted. The simulated collagen loss therefore reflects enzymatic degradation rather than mechanically driven crack growth. Accounting for dynamic lesion evolution would require implementing a damage or fracture mechanics framework to capture material loss and crack propagation under continuous loading^19^—an important but distinct modeling challenge beyond the scope of the current study.

**Authors’ actions:**

*Methods* – *(page 10, lines 183-186):* “A 2D geometry of cartilage explant was created with same dimensions as in the biomechanical model. We did not model lesion formation or crack propagation in the mechanical model, but lesion geometry was added to the biochemical model geometry by segmenting the lesion geometry from one histological section (Figure 1d). The lesion geometry was created to visually assess the localization of collagen loss around this region after injury.”

**Reviewer comment #4:** The current model uses only two time points, Day 0 and Day 12. However, additional time points would offer more dynamic information for better modeling for disease progression as other investigators published models on different time scales.

**Authors’ response:** We thank the reviewer for this valuable suggestion. We agree that additional time points would improve temporal resolution and further strengthen model calibration. Additional time points would greatly improve our understanding of the temporal progression of injury-induced MMP catabolic activity and subsequently collagen loss. Unfortunately, experimental data beyond day 12 were not available for this study, and earlier samples have not yet been analyzed. As described in our response to Reviewer 1 (Comment #2), we are currently working to include intermediate time points (i.e., day 3 and day 7) in future iterations of the model to refine temporal calibration and validation.

**Authors’ actions:**

*Discussion* – *(pages 22-23, lines 398–403):* “… Therefore, the experimental findings and model parameter values presented here might not be directly translatable to human cartilage without further validation. In addition, the collagen content experimental data used in this work for comparison were available only at two time points (i.e., day 0 and day 12). Incorporating intermediate time points in future work would enable a more accurate characterization of the temporal progression of MMP upregulation and catabolic activity following injury-induced cell damage and thereby allow a more precise temporal assessment of collagen loss over the 12-days period.”

**Reviewer comment #5:** The current model reflects only isolated pieces of cartilage. The authors acknowledge the role of cytokines in promoting MMP production in the Discussion section. However, there are distinct possibilities that inflammatory cytokines may have MMP-independent effects on osteoarthritis progression in vivo. The authors should discuss the limitations of the current model.

**Authors’ response:** We agree with the reviewer that inflammatory cytokines can contribute to cartilage degeneration through multiple mechanisms beyond mechanically triggered MMP release and the resulting collagen degradation, including for example the hindering of matrix biosynthesis, increased oxidative stress and cell damage and death.

**Authors’ actions:**

*Discussion* – *(page 23, lines 408–410):* “We acknowledge that, in the context of traumatic joint injuries, collagen fibrils degeneration in cartilage may also be influenced by inflammatory responses originating from surrounding joint tissues, such as the synovium^20–22^. Injury to these tissues can trigger elevated expression of cytokines such as IL-1 and IL-6^20,23^, which may subsequently infiltrate the cartilage and stimulate MMP production^7^. Moreover, these cytokines can exacerbate degradation of collagen fibrils through additional MMP-independent pathways, such as suppressing collagen and aggrecan biosynthesis, increasing oxidative stress and promoting cell apoptosis.”

**References:**

1.	Hosseini, S. M., Wilson, W., Ito, K. & Van Donkelaar, C. C. A numerical model to study mechanically induced initiation and progression of damage in articular cartilage. *Osteoarthritis Cartilage* **22**, 95–103 (2014).

2.	Mononen, M. E., Tanska, P., Isaksson, H. & Korhonen, R. K. A novel method to simulate the progression of collagen degeneration of cartilage in the knee: Data from the osteoarthritis initiative. *Sci Rep* **6**, (2016).

3.	Párraga Quiroga, J. M., Wilson, W., Ito, K. & van Donkelaar, C. C. The effect of loading rate on the development of early damage in articular cartilage. *Biomech Model Mechanobiol* **16**, 263–273 (2017).

4.	Faisal, T. R., Adouni, M. & Dhaher, Y. Y. Surrogate modeling of articular cartilage degradation to understand the synergistic role of MMP-1 and MMP-9: a case study. *Biomech Model Mechanobiol* **22**, 43–56 (2023).

5.	Tanska, P., Julkunen, P. & Korhonen, R. K. A computational algorithm to simulate disorganization of collagen network in injured articular cartilage. *Biomech Model Mechanobiol* **17**, 689–699 (2018).

6.	Rahman, M. M., Watton, P. N., Neu, C. P. & Pierce, D. M. Predicting the heterogeneous chemo-mechano-biological degeneration of cartilage using 3-D biphasic finite elements. *Comput Methods Programs Biomed* **270**, (2025).

7.	Kar, S. *et al.* Modeling IL-1 induced degradation of articular cartilage. *Arch Biochem Biophys* **594**, 37–53 (2016).

8.	Gierig, M., Wriggers, P. & Marino, M. Computational model of damage-induced growth in soft biological tissues considering the mechanobiology of healing. *Biomech Model Mechanobiol* **20**, 1297–1315 (2021).

9.	Eskelinen, A. S. A. *et al.* Mechanobiological model for simulation of injured cartilage degradation via proinflammatory cytokines and mechanical. *PLoS Comput Biol* **16**, (2020).

10.	Taffetani, M., Griebel, M., Gastaldi, D., Klisch, S. M. & Vena, P. Poroviscoelastic finite element model including continuous fiber distribution for the simulation of nanoindentation tests on articular cartilage. *J Mech Behav Biomed Mater* **32**, 17–30 (2014).

11.	Wilson, W., Van Donkelaar, C. C., Van Rietbergen, B., Ito, K. & Huiskes, R. Stresses in the local collagen network of articular cartilage: A poroviscoelastic fibril-reinforced finite element study. *J Biomech* **37**, 357–366 (2004).

12.	Kapitanov, G. I., Ayati, B. P. & Martin, J. A. Modeling the effect of blunt impact on mitochondrial function in cartilage: Implications for development of osteoarthritis. *PeerJ* **2017**, (2017).

13.	Kapitanov, G. I., Wang, X., Ayati, B. P., Brouillette, M. J. & Martin, J. A. Linking cellular and mechanical processes in articular cartilage lesion formation: A mathematical model. *Front Bioeng Biotechnol* **4**, (2016).

14.	Rahman, M. M., Watton, P. N., Neu, C. P. & Pierce, D. M. A chemo-mechano-biological modeling framework for cartilage evolving in health, disease, injury, and treatment. *Comput Methods Programs Biomed* **231**, (2023).

15.	Hamada, M. *et al.* Loss of collagen content is localized near cartilage lesions on the day of injurious loading and intensified on day 12. *Journal of Orthopaedic Research* https://doi.org/10.1002/jor.25975 (2024) doi:10.1002/jor.25975.

16.	Jönsson, V. *et al.* Influence of articular cartilage sample geometry on mechanical response and properties using finite element simulation. *J Biomech* **176**, (2024).

17.	Orozco, G. A., Tanska, P., Florea, C., Grodzinsky, A. J. & Korhonen, R. K. A novel mechanobiological model can predict how physiologically relevant dynamic loading causes proteoglycan loss in mechanically injured articular cartilage. *Sci Rep* **8**, (2018).

18.	Han, E., Chen, S. S., Klisch, S. M. & Sah, R. L. Contribution of proteoglycan osmotic swelling pressure to the compressive properties of articular cartilage. *Biophys J* **101**, 916–924 (2011).

19.	Orozco, G. A., Tanska, P., Gustafsson, A., Korhonen, R. K. & Isaksson, H. Crack propagation in articular cartilage under cyclic loading using cohesive finite element modeling. *J Mech Behav Biomed Mater* **131**, (2022).

20.	Davidson, R. K. *et al.* Expression profiling of metalloproteinases and their inhibitors in synovium and cartilage. *Arthritis Res Ther* **8**, (2006).

21.	Sui, Y. *et al.* Mechanical injury potentiates proteoglycan catabolism induced by interleukin-6 with soluble interleukin-6 receptor and tumor necrosis factor α in immature bovine and adult human articular cartilage. *Arthritis Rheum* **60**, 2985–2996 (2009).

22.	Dayer, J. M. The pivotal role of interleukin-1 in the clinical manifestations of rheumatoid arthritis. *Rheumatology* **42**, (2003).

23.	Redondo, M. L., Christian, D. R. & Yanke, A. B. The Role of Synovium and Synovial Fluid in Joint Hemostasis. in *Joint Preservation of the Knee* 57–67 (Springer International Publishing, Cham, 2019). doi:10.1007/978-3-030-01491-9_4.
